# Assessing reliability of protein-protein interactions by integrative analysis of data in model organisms

**DOI:** 10.1186/1471-2105-10-S4-S5

**Published:** 2009-04-29

**Authors:** Xiaotong Lin, Mei Liu, Xue-wen Chen

**Affiliations:** 1 Bioinformatics and Computational Life-Science Laboratory, ITTC, Department of Electrical Engineering and Computer Science, The University of Kansas, 1520 west 15^th ^Street, Lawrence, KS 66045, USA

## Abstract

**Background:**

Protein-protein interactions play vital roles in nearly all cellular processes and are involved in the construction of biological pathways such as metabolic and signal transduction pathways. Although large-scale experiments have enabled the discovery of thousands of previously unknown linkages among proteins in many organisms, the high-throughput interaction data is often associated with high error rates. Since protein interaction networks have been utilized in numerous biological inferences, the inclusive experimental errors inevitably affect the quality of such prediction. Thus, it is essential to assess the quality of the protein interaction data.

**Results:**

In this paper, a novel Bayesian network-based integrative framework is proposed to assess the reliability of protein-protein interactions. We develop a cross-species *in silico *model that assigns likelihood scores to individual protein pairs based on the information entirely extracted from model organisms. Our proposed approach integrates multiple microarray datasets and novel features derived from gene ontology. Furthermore, the confidence scores for cross-species protein mappings are explicitly incorporated into our model. Applying our model to predict protein interactions in the human genome, we are able to achieve 80% in sensitivity and 70% in specificity. Finally, we assess the overall quality of the experimentally determined yeast protein-protein interaction dataset. We observe that the more high-throughput experiments confirming an interaction, the higher the likelihood score, which confirms the effectiveness of our approach.

**Conclusion:**

This study demonstrates that model organisms certainly provide important information for protein-protein interaction inference and assessment. The proposed method is able to assess not only the overall quality of an interaction dataset, but also the quality of individual protein-protein interactions. We expect the method to continually improve as more high quality interaction data from more model organisms becomes available and is readily scalable to a genome-wide application.

## Background

Protein-protein interactions (PPI) are the foundation of most biological mechanisms such as DNA replication and transcription, enzyme-mediated metabolism, signal transduction, and cell cycle control [1,2]. Therefore, information on the physiological interactions of proteins is perhaps one of the most valuable resources from which annotations of genes and proteins can be discovered. Traditional biology approach studies protein-protein interactions individually by low-throughput technologies [3,4]. In more recent "high-throughput" view, protein interactions are visualized as a sophisticated network and studied globally with technologies such as yeast two-hybrid system [5], affinity purification followed by mass spectrometry [6,7], protein chips [8], gel-filtration chromatography [9], and phase display [10]. These high-throughput genome-wide protein interaction screens have been carried out in many organisms and produced thousands of experimentally identified protein-protein interactions. One major issue, however, is the prevalence of spurious interactions in the high-throughput interaction data. Errors may arise from a wide range of affinities and timescales by which proteins interact with one another. Analysis by Deane *et al*. [11] suggests that only 30–50% of the high-throughput interactions are biologically relevant. In an independent study, Mrowka *et al*. [12] observed significant difference in individually identified interactions from those by genome-wide scans, and estimated that some whole-genome scans may contain 44–91% of false positives. These false positives, i.e. interactions that are detected in the experiment but never take place in the cell, may connect unrelated proteins in the interaction network, create unnecessary interaction clusters, and incorrect biological conclusions may be drawn as a consequence. Hence, to effectively use the high-throughput data in biological inferences, it is critical to evaluate the quality of the data and remove as many false positive interactions as possible.

Various approaches have been proposed to analyze the proteomics data by extracting the subset of valid interactions from their background noise. In some original high-throughput experiments [6,7,13,14], promiscuity criteria are employed to remove proteins having many interaction partners. One limitation of this method is that it can only be applied *ad hoc *because there is no clear separation between the 'sticky' (highly connected) and 'non-sticky' (sparsely connected) proteins. Moreover, biological networks are scale-free in nature [15-19], which implies that the highly connected proteins may as well be a real feature of the protein interaction networks. On the other hand, two independent analyses by von Mering *et al*. [20] and Bader and Hogue [21] studied intersections between different high-throughput datasets and demonstrated that interaction pairs identified by multiple experiments are enriched in true interactions. A shortcoming of this method is the lack of overlap between datasets. Not only data from different technologies do not overlap significantly, but also data from different labs using the same technology differ substantially. This suggests that the current data are far from saturating, and data from different resources are complementary to each other.

It is also possible to explore the relationship between protein-protein interaction data and other types of data to assess the quality. Mrowka *et al*. [12] compared distributions of transcription correlations between the interaction data from many single hypothesis-driven experiments and genome-wide scans. Using data from the Munich Information Center for Protein Sequences (MIPS) [22] as the reference set of true interactions, they described a bootstrap method to count how many random pairs needed to be inserted in order to create the same statistical behaviour of the expression correlation as in the putative interaction data. Other colleagues applied microarray and mRNA expression data to assess the quality of protein-protein interaction data [11,23,24]. Nevertheless, interacting proteins do not necessarily display correlation in mRNA levels. In fact, proteins in a permanent complex may even show low transcriptional correlation due to differences in degradation rates [25]. Even worse, Bader *et al*. [26] noticed that for the data from mass spectrometry of coimmunoprecipitated protein complexes (Co-IP), the correlated coexpression may be negatively correlated with predicted interaction confidence.

Besides expression data, sequence homology between two proteins and their corresponding interaction partners has been adopted to verify high-throughput protein-protein interactions [11]. However, the verification process is restricted to interaction pairs with both proteins having homologs, and even for these applicable interaction pairs, only half are identified as high confident under the homology criterion [11]. Moreover, other groups made use of cellular localization and cellular-role properties to assess the reliability of high-throughput experimental data [20,24,27]. Furthermore, Saito *et al*. [28] and Goldberg and Roth [29] exploited network topological descriptors to determine how well an edge (interaction) fits the expected topology of protein-protein interaction network. Altogether, the aforementioned methods apply threshold values to assess the quality of interactions by classifying them as either high or low confidence. Likewise, a number of computational approaches for protein interaction prediction have been developed to designate two proteins as either interacting or not interacting based on genomic context [30-37] and protein domain [38-43]. Despite their varying successes, it is much more beneficial to estimate the probability that a pair of proteins may form a true interaction rather than producing a binary outcome.

Recently, there has been a growing interest in data integration. In a study on the yeast signal transduction pathway for amino acid transport, Chen and Xu [44] demonstrated that integration of high-throughput data with other biology resources can transform the noisy protein interaction data into useful knowledge. Many probabilistic methods have explored the integration of complementary data sources for protein interaction inference, which turned out to improve both accuracy and coverage. Integrating diverse types of evidences such as gene expression, gene ontology (GO) [45], and enriched domain pairs, research groups have proposed probabilistic decision tree [46], logistic regression [26,47], naïve Bayes [48,49], and Bayesian network [29,50] models.

In this study, we describe a novel Bayesian network-based integrative model that assigns a likelihood score to every interaction. The main contributions we make are as follows. First, we establish a cross-species *in silico *model to assess confidence of two proteins to interact in a target organism (e.g. human) on the basis of information entirely extracted from other model organisms (e.g. *Saccharomyces cerevisiae*, *C. elegans*, and *Drosophila melanogaster*). A cross-organism computational system for protein interaction prediction is attractive and needed, mainly because model organisms are well studied and have a tremendous amount of experimental data, while there may be little information about the target organism, especially with newly sequenced proteins (thus, prediction based on the target organism may be impossible or inaccurate due to data scarcity). Among protein interaction studies using data from model organisms, data from target organism is employed in addition to data from other organisms [47,49]. In existing integrative models, data from model organisms may not even play a significant role. For instance, Rhodes *et al*. [49] showed that information from model organisms alone is only moderately predictive. Thus, there is an essential need for better probabilistic models that can effectively integrate heterogeneous data sources from model organisms. Our proposed model demonstrates that a carefully designed system is capable of making accurate assessment utilizing information solely from model organisms. Second, we introduce a novel Bayesian network-based approach to integrate multiple microarray datasets and GO information. In contrary to commonly used naïve Bayes model, we do not make conditional independence assumption among multiple microarray datasets and new features extracted from GO biological processes, molecular functions, and cellular components. Furthermore, the confidence scores for orthologous mappings are explicitly incorporated into our model. Finally, applying our cross-species *in silico *model, we assess the overall quality of the protein-protein interaction data obtained from high-throughput screens for yeast.

## Results and discussion

### System overview

**Figure 1:**
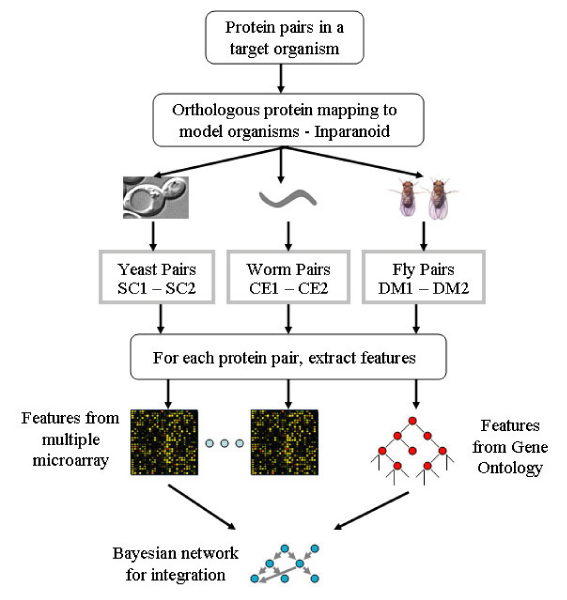
**System overview**. Predict protein-protein interactions in a target organism using information extracted from model organisms. Heterogeneous data are integrated by a Bayesian network-based method.

The proposed cross-organism predictive system is illustrated in Figure 1. For a pair of proteins (*P*_1_, *P*_2_) in a target organism, genome-wide orthologous mapping between the target organism and model organisms can be obtained from the InParanoid database [51]. The InParanoid program uses NCBI-BLAST to calculate the pairwise similarity scores between two complete proteomes. A confidence value *C *is then provided to evaluate how closely related two orthologs are.

Our strategy is as follows. First, for a pair of proteins (*P*_1_, *P*_2_), we determine their orthologs in the model organisms. Second, features are extracted for each ortholog pair from gene expression profiles and GO annotations of model organisms (details are discussed in next section). Finally, the heterogeneous data features are integrated to describe the protein pair (*P*_1_, *P*_2_) of the target organism using a Bayesian network-based model (details are in Methods) that assigns likelihood ratios for interaction.

### Novel feature extraction

**Figure 2:**
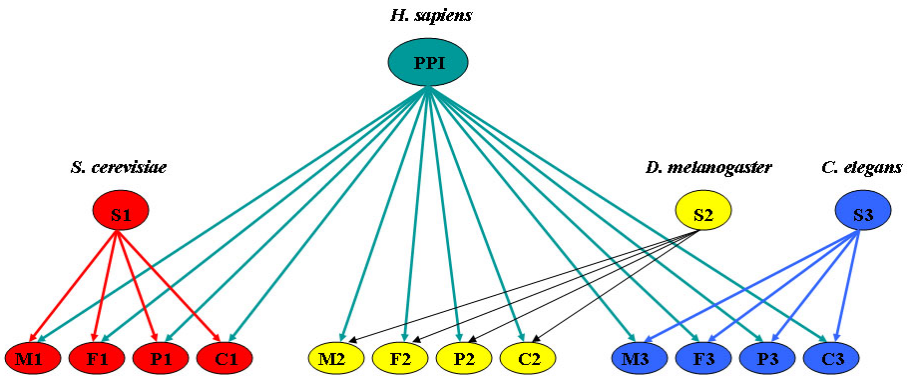
**Bayesian network-based framework**. Integrates heterogeneous data sources from model organisms (*S. cerevisiae*, *D. melanogaster*, and *C. elegans*) to predict protein-protein interactions in a target organism (*H. sapiens*).

To determine how likely two proteins will interact, several features are derived from gene expression profiles and GO annotations. For each protein pair (*P*_1_, *P*_2_) in the target organism, we identify its ortholog pairs (*R*_(*i*)*1*_, *R*_(*i*)*2*_) in three model organisms (*i *= 1, 2, 3). From each model organism, we download three microarray datasets. For each ortholog pair (*R*_(*i*)1_, *R*_(*i*)2_), Pearson correlation coefficients (PCC) are calculated from the gene expression profiles. A 4-level uniform quantization is used and each PCC is discretized into one of four states: high, medium high, medium low, and low. Rather than assuming the PCCs extracted from different microarray data are independent, we model the three PCCs from individual model organism jointly with one node in our Bayesian network model (Figure 2). This is very different from the commonly-used naïve Bayes method in which every feature is assumed to be independent of each other.

**Figure 3:**
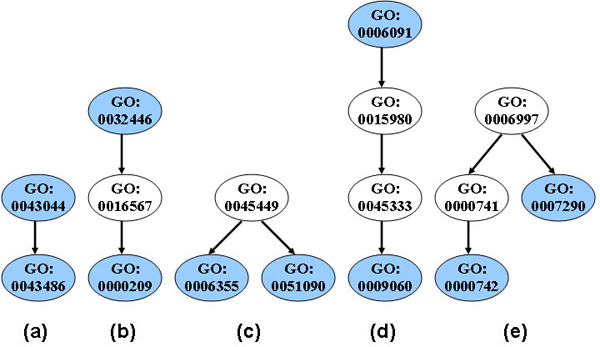
**GO distance structures**. The GO terms in blue circles represent the terms under consideration (i.e. term_3 and term_4 in text) that are the closest in terms of GO distance *d*. (a) *d *= 1 and feature value = 1, GO term_3 is parent of term_4; (b) *d *= 2 and feature value = 2, term_3 is term_4's grandparent or ancestor; (c) *d *= 2 and feature value = 3, term_3 and term_4 share a common parent; (d) *d *= 3 and feature value = 4, term_3 is term_4's great grandparent or ancestor (e) *d *= 3 and feature value = 5, term_3's parent and term_4 are siblings sharing the same parent.

Moreover, we derive novel features from GO annotations for each identified ortholog pair (*R*_(*i*)*1*_, *R*_(*i*)*2*_) of the protein pair (*P*_1_, *P*_2_). Three unique features are derived from each of the "molecular function", "biological process", and "cellular component" annotations in GO. The first feature checks whether two proteins share annotation terms: if the two proteins share at least one common term, the feature value is one; otherwise, it is zero. The second feature is called correlation ratio. In GO, gene products can be associated with more than one term. Therefore, the correlation between two GO terms is defined as the number of gene products in common. The larger the correlation value is, the closer the two GO terms are. We examine all possible pairs of GO terms between the two proteins and identify two GO terms (we refer them as "term_1" and "term_2") with the largest correlation value. The correlation ratio is then defined as *n/(n*_1_+ *n*_2_- *n)*, where *n *is the correlation between term_1 and term_2, *n*_1 _and *n*_2 _are the numbers of gene products with term_1 and term_2, respectively. The correlation ratio is also quantized into two levels with a threshold of 0.5: high and low. The third feature is based on the minimum GO distance *d *between two proteins. Since GO is organized as a directed acyclic graph where each node represents a GO term, distance between two terms is described as the least number of nodes separating them in the graph. Again, to identify the two GO terms ("term_3" and "term_4") with the minimum distance, we examine all possible pairs of GO terms between the two proteins. For the third feature, incorporating the graph structures, we define eight states: 0 if *d *is zero; 1 if *d *is one (Figure 3a); 2 if *d *is two and term_3 and term_4 have a grandparent-children relationship (Figure 3b); 3 if *d *is two and term_3 and term_4 are siblings with a common parent term (Figure 3c); 4 if *d *is three and term_3 is term_4's great grandparent or vice versa (Figure 3d); 5 if *d *is three and term_3's parent and term_4 are siblings with a common parent term (Figure 3e); 6 if *d *is three with all remaining graph structural cases; and 7 if *d *is larger than three. Apparently, the three features are not independent to each other. In our integrative system, we model the three features from each model organism jointly with one node.

### Human protein-protein interaction prediction

It is important to investigate how widely applicable our approach is for automatic verification of large sets of interactions. If a method is sufficient, its predicted protein-protein interactions (PPIs) should have higher overlap with the previously established interactions. To evaluate our integrative method, we use both specificity and sensitivity. The specificity is defined as the percentage of matched non-interactions between the predicted set and the observed set over the total number of observed non-interactions. The sensitivity is defined as the percentage of matched interactions over the total number of observed interactions.

First of all, with a specificity of 95%, our method can achieve a sensitivity of about 44%, and if the specificity reduces to 50%, the sensitivity increases to 80%. These results clearly demonstrate that model organisms certainly provide significant information for the prediction of PPIs in the target organisms. Secondly, we compare our method to the commonly-used naïve Bayesian method [48,49]. In the naïve Bayesian model, all the features are assumed to be conditionally independent, i.e., features extracted from three microarray data sets and three novel features from GO in each model organism are conditionally independent given PPI. Table 1 contains results of our method and the naïve Bayesian method over the test dataset. With comparable specificities fixed at approximately the same level 70%, our method can achieve 73% in sensitivity and the naïve Bayesian can only reach 65% in sensitivity.

**Table 1:** Accuracy comparison. Our method (I): results on all test data (with at least one model organism). Our method (II): results on test data with orthologous mapping from three model organisms

	**Naïve Bayesian**	**Our method (I)**	**Our method (II)**
Sensitivity	65%	73%	80%
Specificity	70%	70%	70%

**Figure 4:**
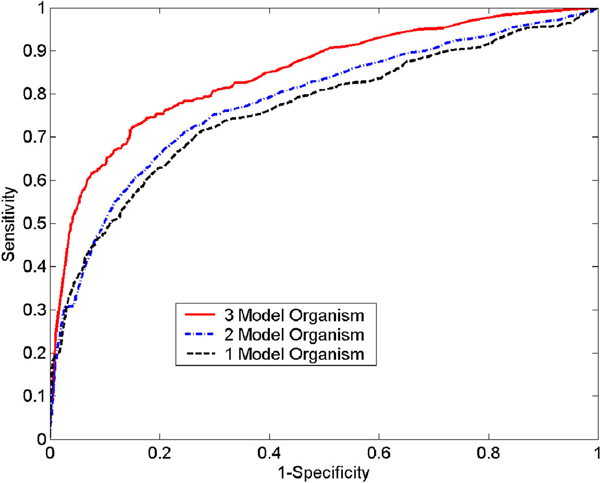
**ROC curves with different number of model organisms**. Illustrates the ROC curves for test data with mapping orthologs from one, two, and three model organisms.

Figure 4 illustrates the ROC curves for the test data with mapping orthologs from one, two, and three model organisms. As expected, the system performance increases as more evidence is available. With mapping information available from three organisms, we can achieve 70% in specificity with a sensitivity of 80% (Table 1). Thus, the method will continue to improve as more interaction data from more model organisms becomes available.

### Assessment of yeast protein-protein interaction data

While high-throughput technologies generate thousands of protein-protein interactions (PPI) data and allow for genome-wide analysis, they tend to produce a large number of false positives. On the other hand, low-throughput methods can yield reliable results but are typically labor intensive, time consuming, and on a small scale basis. Computational methods provide an ideal tool for evaluating experimentally detected PPIs, as *in silico *methods can (1) utilize existing biological knowledge; (2) predict large-scale PPIs; and (3) produce the confidence levels of interactions for each protein pairs.

We apply our cross-organism integrative *in silico *model to evaluate high-throughput yeast PPI data and detect the spurious interactions. Our model is ideal for this type of application, as we do not use the direct PPI data from model organisms in our training process (features are extracted from microarray data and GO only). The current available yeast interaction pairs in databases may be determined by various experiments; therefore, the more experiments confirming it, the more confident we are in the interaction.

We collected the yeast interaction data from the General Repository for Interaction Datasets (BioGRID) [52]. The deposited interactions are determined through a number of methods, but we mainly focus on four: synthetic lethality, affinity capture-MS, two-hybrid, and phenotypic enhancement. Total number of PPIs detected by each of the experimental methods are 9378, 24154, 7157, and 15815 for synthetic lethality, affinity capture-MS, two-hybrid, and phenotypic enhancement, respectively. Among the four datasets, total number of unique pairs is 52783. Therefore, there are 3739 overlapping pairs between the datasets. Because our goal is to analyze the system on PPIs determined by different number of experiments, four data files are generated, in which one contains interaction pairs identified by only one experiment, another contains pairs from only two experiments, etc. Finally, there are 49260, 3333, 182, and 8 PPIs identified by one, two, three, and four different experiments, respectively.

**Figure 5:**
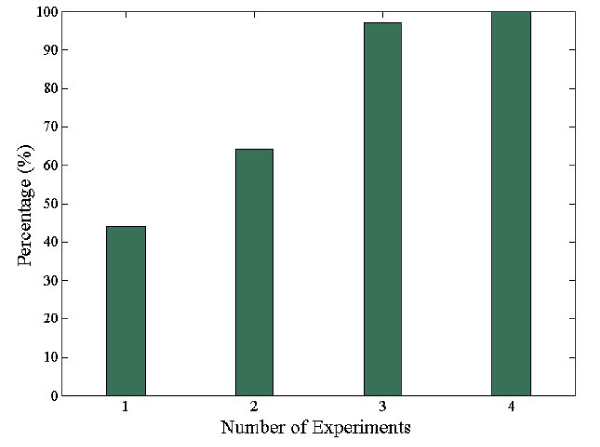
**Percentage of PPIs detected by our method**. Shows the percentage of PPIs detected by different number of high-throughput experiments that are also predicted by our cross-organism integrative model.

Each PPI pair can be ranked by the likelihood ratio (positive versus negative). The larger the ratio is, the higher confidence we have in the interactions. We consider a protein pair as interacting if its likelihood ratio is larger than one (i.e., the likelihood of "interaction" is larger than that of "non-interaction"). Figure 5 shows the percentage of PPIs detected by high-throughput methods that are also predicted by our cross-organism model. As can be seen, all the PPIs that are supported by four different experiments are also predicted as positives by our model. We also predict 44%, 64%, and 97% of PPIs detected by one, two, and three biological experiments as interacting protein pairs, respectively. Notably, the percent of true positives (as verified by our model) for PPIs with only one experimental evidence is similar to the positive rate estimated by Sprinzak *et al*. [27].

**Figure 6:**
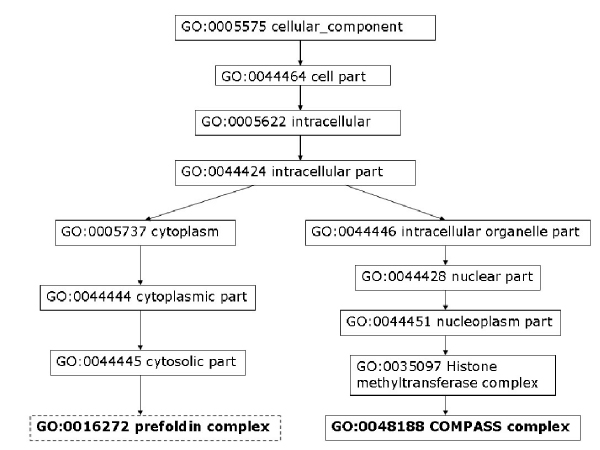
**YJL179W & YBR258C closest cellular component**. Closest GO cellular component terms for protein pair YJL179W – YBR258C. Highlighted GO terms in dashed boxes are annotations for the first protein and ones in solid boxes are for the second protein.

**Figure 7:**
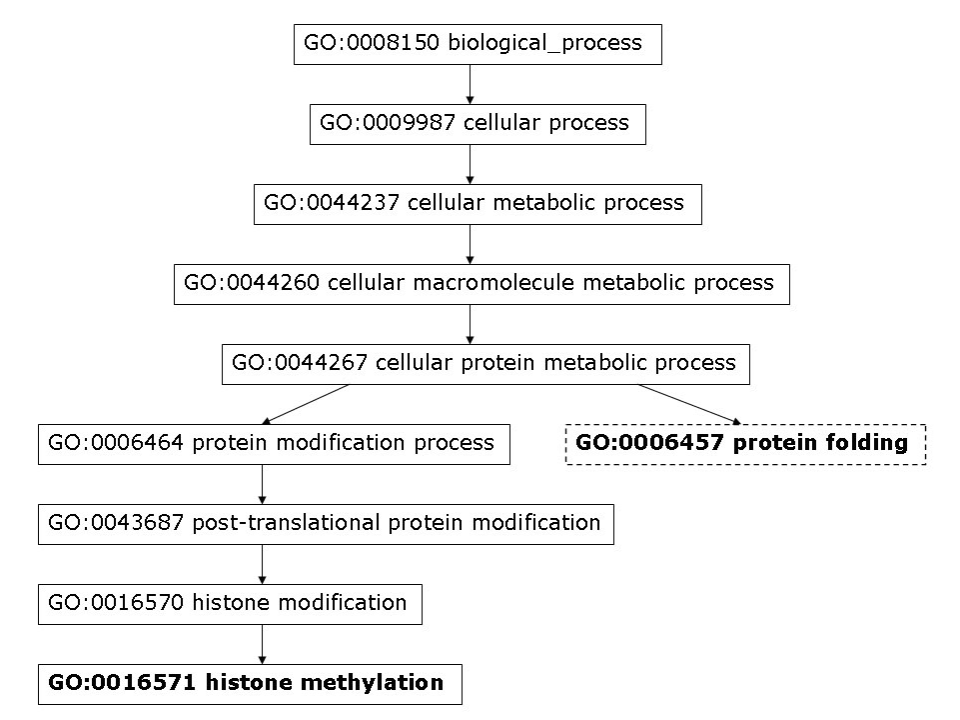
**YJL179W & YBR258C closest biological process**. Closest GO biological process terms for protein pair YJL179W – YBR258C. Highlighted GO terms in dashed boxes are annotations for the first protein and ones in solid boxes are for the second protein.

**Figure 8:**
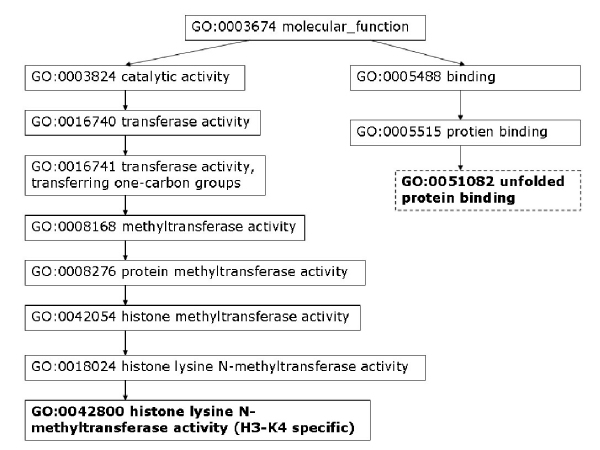
**YJL179W & YBR258C closest molecular function**. Closest GO molecular function terms for protein pair YJL179W – YBR258C. Highlighted GO terms in dashed boxes are annotations for the first protein and ones in solid boxes are for the second protein.

Moreover, we analyze some PPIs detected by high-throughput experiments but predicted as negatives by our model. To assess the data, we consider the shortest distance of two proteins in GO cellular components, molecular function, and biological process. As discussed by Sprinzak *et al*. [27], for true interactions, the interacting proteins should be localized in the same cellular compartment or participate in the same cellular process. The protein pair, YJL179W and YBR258C, is identified by one high-throughput experiment but predicted as non-interacting by our method. The closest cellular component terms between YJL179W and YBR258C are GO:0016272 (a multisubunit chaperone that acts to deliver unfolded proteins to cytosolic chaperonin that resides in the cell cytoplasm) and GO: 0048188 (a conserved protein complex that catalyzes methylation of histone H3, which belongs to the nucleoplasm part). As can be seen in Figure 6, these two proteins are not localized to the same compartment. We can also observe that the two proteins do not participate in the same cellular process (Figure 7) nor execute the same function (Figure 8).

**Figure 9:**
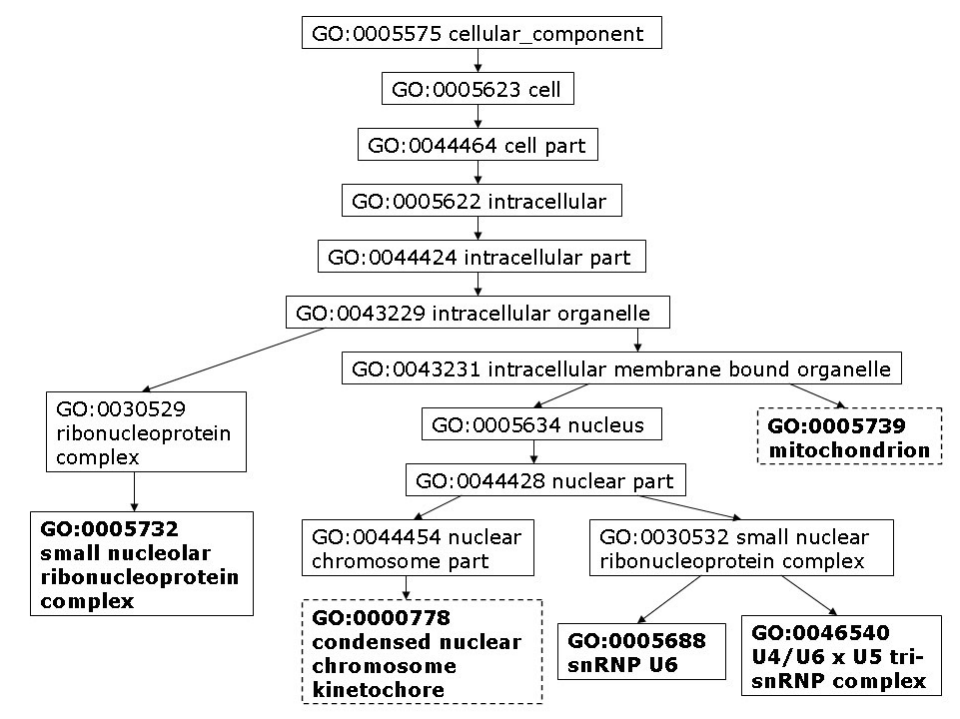
**YEL061C & YNL147W closest cellular component**. Closest GO cellular component terms for protein pair YEL061C – YNL147W. Highlighted GO terms in dashed boxes are annotations for the first protein and ones in solid boxes are for the second protein.

**Figure 10:**
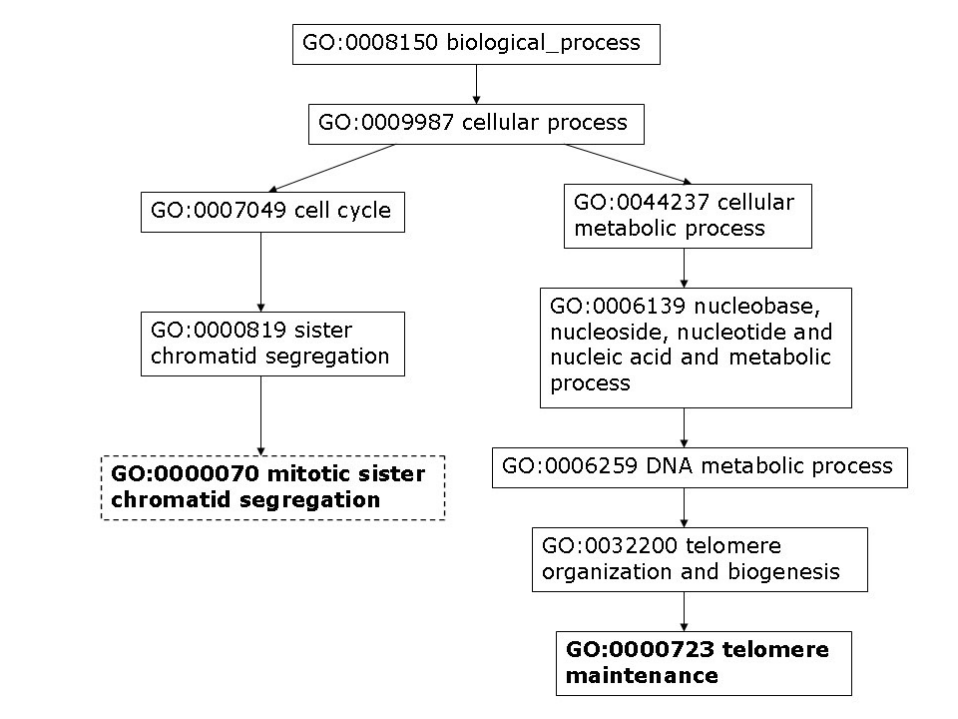
**YEL061C & YNL147W closest biological process**. Closest GO biological process terms for protein pair YEL061C – YNL147W. Highlighted GO terms in dashed boxes are annotations for the first protein and ones in solid boxes are for the second protein.

**Figure 11:**
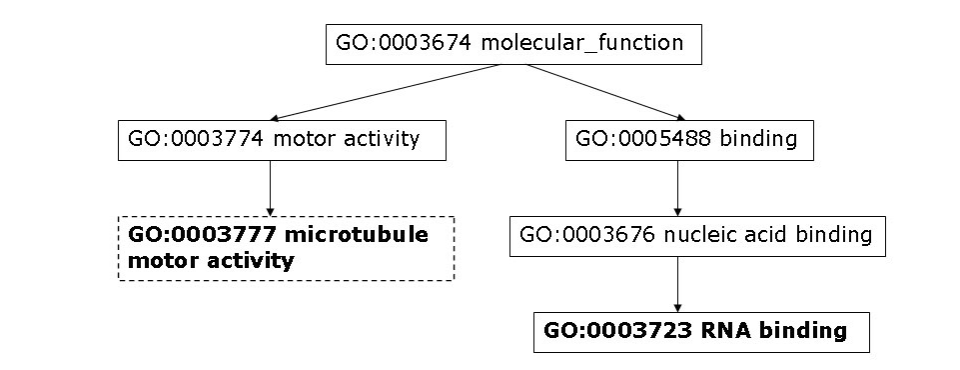
**YEL061C & YNL147W closest molecular function**. Closest GO molecular function terms for protein pair YEL061C – YNL147W. Highlighted GO terms in dashed boxes are annotations for the first protein and ones in solid boxes are for the second protein.

Similar observation can be made regarding protein pairs supported by two biological experiments but predicted as non-interacting. For example, the protein pair, YEL061C and YNL147W, has several pairs of GO cellular component terms that are closest to each other between the two proteins: (GO:0000778, GO:00005732), (GO:0000778, GO:0005688), (GO:0000778, GO:0046540), and (GO:0005739, GO:0005732) (Figure 9). GO:0000778 refers to a multisubunit complex that is located at the pericentric region of a condensed chromosome in the nucleus and provides an attachment point for the spindle microtubules. GO:0005739 describes a semiautonomous, self replicating organelle that occurs in varying numbers, shapes, and sizes in the cytoplasm of virtually all eukaryotic cells. It is notably the site of tissue respiration. GO:0005732 represents a complex composed of RNA of the small nucleolar RNA (snoRNa) and protein, found in the nucleolus of a eukaryotic cell. GO:0005688 refers to the ribonucleoprotein complex containing small nuclear RNA U6; a component of the major spliceosome complex. GO:0046540 refers to a complex composed of three small nuclear ribonucleoproteins, snRNP U4, snRNP U6, and snRNP U5. Figures 10 and 11 illustrate the closest biological process and molecular function terms, respectively.

## Conclusion

The advent of high-throughput technologies has significantly enlarged the collection of protein-protein interactions. On one hand, it has provided a rich source of information for new biological discoveries. On the other hand, it has introduced a technical challenge due to its high error rates. It has been shown by many researchers that the reliability of high-throughput screens is only about 50%. The large number of false positives may result in false biological conclusions. It is thus essential to assess the quality of the interactions.

In this paper, we develop a novel Bayesian network-based model that integrates heterogeneous data sources from model organisms to determine the probability of two proteins to interact in a target organism. Cross-species prediction is attractive as normally we do not have much information about newly sequenced proteins. By mapping them to well studied model organisms; however, we are able to utilize the existing biological knowledge of the model organisms to make accurate predictions. Our model is successfully applied to predict protein-protein interactions in human. For the protein pairs with orthologous mappings in all three model organisms, our model can achieve 80% in sensitivity and 70% in specificity. The method is also successfully applied to assess the quality of the high-throughput interaction data. We observed that the more high-throughput experiments confirming an interaction, the higher the confidence score is assigned by our method. For the protein pairs confirmed by four different biological experiments, we predicted all of them as interacting. For the pairs supported by only one experiment, the percentage of true positives we determined is similar to the positive rate estimated by Sprinzak *et al *[27].

The above results demonstrate that model organisms indeed provide important information for protein-protein interaction inference and assessment. The method is able to assess not only the overall quality of an interaction dataset, but also the quality of individual protein-protein interactions. We expect the method to continually improve as more high quality interaction data from more model organisms becomes available and is readily scalable to a genome-wide application.

## Methods

### Data collection

The interaction data for *S. cerevisiae*, *C. elegans*, and *D. melanogaster *are collected from the General Repository for Interaction Datasets (BioGRID) [52]. In total, we gathered 4,433 *C. elegans*, 33,518 *D. melanogaster*, and 111,611 *S. cerevisiae *interaction pairs. The human interacting protein pairs are obtained from the Human Protein Reference Database (HPRD) [53,54] where the data is manually curated by expert biologists. From the HPRD, we acquired total 30,819 human interaction pairs. As our model is a cross-species model, protein pairs without orthologous mappings in any model organisms need to be excluded. Finally, we end up with 10,163 human interaction pairs as our positive data. Since the negative or non-interacting protein data is not available, we randomly generate the negative samples. A protein pair is considered to be a negative sample if the pair does not appear in the existing interaction dataset. Total of 209,761 negative samples are generated. The ratio of negatives and positives is about 20:1. About 2/3 of positive and negative data are reserved as training data and the remaining samples are used as testing data. The final training set has 6,766 positive pairs and 139,864 negative pairs, and the testing set contains 3,397 positives and 69,897 negatives.

Genome-wide orthologous mapping between the target organism and model organisms is obtained from the InParanoid database [51]. InParanoid determines protein mappings by constructing a protein cluster using a reciprocally best-matching ortholog pair as seed, and inparalogs are gathered independently around the seed ortholog pair. Each member of the cluster receives an inparalog score between 0 and 1.0, which reflects the relative distance to the seed-inparalog. This inparalog score is regarded as the orthologous mapping confidence score in this paper. For each protein pair in human, our target organism, we form a list of ortholog pairs in the model organisms. Then, for each of those ortholog pairs, we combine microarray gene expression data and Gene Ontology (GO) information to estimate the probability that two proteins interact in the target organism. From GO, we retrieve 'molecular function', 'biological process', and 'cellular component' annotations for each protein under consideration.

Microarray gene expression data are collected from NCBI Gene Expression Omnibus (GEO) [55,56]. Only datasets with more than 20 samples are selected. We downloaded three microarray datasets for each model organism as shown in Table 2 (yeast [57-59]; worm [60-62]; fruit fly [63,64]).

**Table 2:** Microarray datasets used in our experiment. N is the number of samples in each data set.

**Organism**	**Dataset (N)**	**Dataset (N)**	**Dataset (N)**
Yeast	GDS1115 (131)	GDS465 (90)	GDS92 (40)
Worm	GDS1319 (123)	GDS770 (20)	GDS6 (29)
Fruit fly	GDS2272 (36)	GDS516 (26)	GDS2673 (27)

### Integrative model

The heterogeneous data from different organisms are integrated using a Bayesian network (BN) model as shown in Figure 2. Bayesian network is a graphical model that encodes the probabilistic dependencies among a set of variables [65]. It consists of two important components: a directed acyclic graph (DAG) representing the dependency structure among the variables in the network and a conditional probability table or a distribution for each variable in the network given its parent set [66]. Our first application is to predict protein-protein interactions (PPI) of *H. sapiens *by integrating information from three model organisms (*S. cerevisiae*, *C. elegans*, and *D. melanogaster*) as shown in Figure 2. The node PPI is a binary variable representing the class membership: two human proteins will be predicted to interact with each other if PPI = 1 or form non-interaction if PPI = 0. Variables S1, S2, and S3 represent three model organisms and are ternary: 2, 1, and 0 indicate a strong, medium, or weak confidence of the orthologous mapping in terms of the confidence value *C *from the InParanoid, respectively. Thus, the confidence scores of mapping for each ortholog set are explicitly incorporated into our model.

From each model organism, we extract microarray features and GO features as discussed above. The nodes *M*_*i*_, *F*_*i*_, *Pi*, and *C*_*i *_represent features extracted from **M**icroarray data, molecular **F**unction, biological **P**rocess, and cellular **C**omponent from model organism *i *(*i *= 1 yeast, 2 fruit fly, 3 worm), respectively. For each model organism, we compute Pearson Correlation Coefficient (PCC) from three microarray datasets and each PCC is discretized into 4 levels (high, medium high, medium low, and low). Unlike the commonly used naïve Bayes model, we do not assume that microarray datasets are conditionally independent. We model them jointly using the node *M*_*i*_, a node with 20 states. For example, *M*_*i *_= 1 or (low, low, low) indicates that the PCC values calculated from the three microarray data sets are all low; *M*_*i *_= 2 or (low, low, medium low) means that PCC values are low in two microarray data sets and medium low in one microarray data set. Note that high-high-low, low-high-high and high-low-high etc. are considered as the same state. In other words, we only consider the PCC levels regardless of which microarray data set is used.

Similarly, *F*_*i*_, *P*_*i*_, and *C*_*i *_represent the combination of three features extracted from GO for each organism. For example, the variable *F*_1 _is a vector of (feature 1: shared function terms, feature 2: correlation ratio, feature 3: GO distance) and has 32 states (two states for features 1 and 2 and eight states for feature 3; refer to the 'Novel Feature Extraction' section for details). We summarize the information for each node in Table 3.

**Table 3:** Information of nodes in Figure 2

**Nodes**	**# of states**	**Description**
PPI	2	PPI interaction in a target organism 1 – interaction; 0 – non-interaction
S1, S2, S3	3	High/medium/low mapping confidence for the three model organisms
M1, M2, M3	20	PCC levels from three microarray data sets for each model organism
F1, F2, F3	32	Each node is a combination of three features extracted from GO molecular function
P1, P2, P3	32	Each node is a combination of three features extracted from GO biological process
C1, C2, C3	32	Each node is a combination of three features extracted from GO cellular component

The BN model integrates heterogeneous data from three model organisms to predict PPIs in a target organism. For each model organism, features extracted from multiple microarray data or GO terms are modelled jointly without assuming conditional independence. Features of different model organisms are conditionally independent giving the interaction information of a protein pair in the target organism and model organisms. This cross-organism conditional independence allows us to derive a simple solution for PPI prediction, as we detail next.

The Bayesian approach to classify a test sample is to assign the most probable class or the class with a larger posterior probability for a two-class problem. Based on Bayes theorem, we can write the posterior probability of PPI given all the evidence *E*_*i *_= *(S*_*i*_, *M*_*i*_, *F*_*i*_, *P*_*i*_, *C*_*i*_*)*, *i *= 1, 2, 3 as

(1) P(PPI|E1,E2,E3)=P(E1,E2,E3|PPI)⋅P(PPI)P(E1,E,2E3)

For the model shown in Figure 2, we have

(2) $$P(E_{1},E_{2},E_{3}|PPI)=\prod_{i=1}^{3}P(S_{i})\cdot P(M_{i},F_{i},P_{i},C_{i}|S_{i},PPI)$$

The ratio of the posterior probability for two classes is

(3) $$L=\frac{P(PPI=1)\prod_{i=1}^{3}P(S_{i})P(M_{i},F_{i},P_{i},C_{i}|S_{i},PPI=1)}{P(PPI=0)\prod_{i=1}^{3}P(S_{i})P(M_{i},F_{i},P_{i},C_{i}|S_{i},PPI=0)}$$

where (based on conditional independence shown in Figure 2)

(4) $$\begin{array}{r} P(M_{i},F_{i},P_{i},C_{i}|S_{i},PPI)=P(M_{i}|S_{i},PPI)P(F_{i}|S_{i},PPI)\\ \times P(P_{i}|S_{i},PPI)P(C_{i}|S_{i},PPI) \end{array}$$

The prior *P*(*PPI *= 1) and *P*(*PPI *= 0) can be computed empirically. In application, we compute probability ratio *L *for a pair of proteins and predict the two proteins as an interacting pair if *L *> 1 and non-interacting pair otherwise. ROC curves are created by varying this decision threshold, which is equivalent to adjusting the priors. The individual likelihood can be computed from training data.

## List of abbreviations used

PPI: Protein-Protein Interactions; GO: Gene Ontology; MIPS: Munich Information Center for Protein Sequences; Co-IP: coimmunoprecipitated protein complex; ROC: Receiver Operating Characteristic; BioGRID: General Repository for Interaction Datasets; HPRD: Human Protein Reference Database; DAG: Directed Acyclic Graph; PCC: Pearson Correlation Coefficient; BN: Bayesian Network

## Competing interests

The authors declare that they have no competing interests.

## Authors' contributions

XTL carried out the experiments and participated in acquisition and preparation of the microarray data. ML participated in acquisition of protein interaction data and carried out feature extraction of the gene ontology data. XWC conceived the study and designed the experiments. All authors helped in drafting the manuscript and approved the final manuscript.
